# Performance and Tactical Indicators in Elite Olympic RS:X Sailing Across Different Wind Conditions Using GPS Data

**DOI:** 10.3390/s26113312

**Published:** 2026-05-23

**Authors:** Javier Riscart-López, Luka Pezelj, Carlos Manuel Pérez-Pérez, Israel Caraballo-Vidal

**Affiliations:** 1 Department of Medicine and Surgery, Faculty of Medicine, University of Cádiz, 11003 Cádiz, Spain; javier.riscart@uca.es; 2 Faculty of Maritime Studies, University of Split, 21000 Split, Croatia; luka.pezelj@pfst.hr; 3 Department of Physical Education, Faculty of Education Sciences, University of Cádiz, 11003 Cádiz, Spain; israel.caraballo@uca.es

**Keywords:** performance, sailing, windsurfing, Olympic, GPS

## Abstract

The variables determining performance under different wind conditions in Olympic RS:X class sailors have not yet been fully clarified. The aim of this study was to examine the relationship between race performance (final ranking) and GPS-derived variables, including velocity made good (VMG), distance covered, and number of maneuvers, under different wind conditions in Olympic RS:X class sailors. Fifty-four Olympic RS:X class sailors (26 women and 28 men) who competed in a World Cup event were analyzed. Using GPS devices, the first-leg ranking, VMG, number of maneuvers, and distance covered in each segment (upwind, beam reach, and downwind) of every race were evaluated under three wind conditions (light wind: ≥8 to ≤12 knots; moderate wind: >12 to ≤15 knots; strong wind: >15 knots). A total of nine races were analyzed for both the men’s and women’s groups. For both groups and under all wind conditions, first-leg ranking was the variable most strongly associated with overall performance. VMG in upwind and downwind legs was among the most decisive variables in moderate wind conditions for both men and women. In strong wind conditions, VMG during the beam reach was a key variable influencing performance in both groups. In the women’s group, regardless of wind speed, VMG during the first upwind leg was the determining factor for achieving a better first-leg position. In the men’s group, particularly in medium wind conditions, a favorable first-leg position was mainly determined by VMG during the first upwind leg.

## 1. Introduction

Windsurfing is a highly demanding discipline that merges the technical principles of sailing with the dynamic balance requirements of board sports. Performance depends on the athlete’s ability to efficiently harness wind energy through a specialized board–rig system, while simultaneously managing substantial physiological and mechanical loads [1]. Within Olympic competition, the RS:X class follows a standardized race format designed to evaluate upwind efficiency, downwind control, and tactical decision-making under variable environmental conditions [2]. Although minor adjustments may occur depending on the venue and wind characteristics, RS:X events typically employ a trapezoid course, a configuration widely adopted in Olympic sailing to ensure consistency and fairness across races [3].

The trapezoid layout consists of four primary marks that structure a sequence of upwind, reaching, and downwind legs. The internal angle is commonly set at 70°, and the reach between Mark 1 and Mark 2 is standardized at 0.5 nautical miles, providing uniform tactical demands across events. Mark 3 is positioned slightly to windward of the start line, approximately 0.05 nm, to prevent athletes from cutting the line while planing at high speeds, a modification specific to RS:X due to the board’s hydrodynamic behavior. The race duration typically ranges from 20 to 25 min, with the total distance varying between 6 and 9 km depending on wind intensity. Most Olympic RS:X races include two full laps followed by a short final leg to the finish [2,3].

The physical and technical requirements of RS:X windsurfing are considerable. Athletes must combine aerobic and anaerobic capacity, isometric strength, and dynamic postural control to maintain optimal board speed and stability across rapidly changing conditions [1]. Physiological studies indicate that elite competitors frequently sustain intensities exceeding 75% of VO_2_max and 85% of maximal heart rate during racing [1]. These demands are largely attributable to the sail-pumping technique, a repetitive, high-frequency action that generates significant mechanical stress on the upper limbs, trunk, and lower extremities, increasing the risk of overuse injuries, particularly in the lumbar region [4]. As a result, high-performance training programs emphasize unilateral coordination, muscular endurance, and the management of concentric loading patterns [1].

In competitive contexts, particularly within the Olympic RS:X class, success depends on a precise understanding of the variables that determine velocity under different wind conditions [5]. The incorporation of global positioning systems (GPS) and global navigation satellite systems (GNSS) has transformed performance analysis in sailing by enabling accurate quantification of external load, kinematic patterns, and tactical behavior [6,7]. High-frequency GPS devices (e.g., 5 Hz) allow a detailed assessment of mean velocity, the distance covered, and temporal efficiency across race segments. Among these metrics, velocity made good (VMG), the effective speed toward the windward or leeward mark, has emerged as one of the most relevant performance indicators, consistently distinguishing elite from sub-elite competitors [8].

RS:X performance results from the interaction of technical, tactical, and physiological factors, with GPS-derived variables such as VMG, mean velocity, and the distance covered serving as key performance indicators (KPIs) [2]. In light-wind conditions, success is strongly associated with the metabolic efficiency of sail pumping to maximize board speed. In contrast, moderate-to-strong winds shift the performance determinants toward maintaining planing conditions, optimizing hydrodynamic efficiency, and executing superior tactical decisions during upwind legs [8]. Previous research highlights that, across wind ranges, the first upwind leg is often the strongest predictor of the final race outcome [2,8,9].

The relevance of early-race positioning is not unique to sailing. In middle-distance running, for example, the first 200 m of an 800 m race are a critical determinant of final classification, with athletes in the leading positions at the break line showing a significantly higher probability of achieving top finishes [10,11,12,13,14]. Early positioning reduces tactical constraints, minimizes unnecessary accelerations, and preserves anaerobic reserves for the final sprint. Conversely, athletes starting from the rear are forced to cover additional distances and expend greater metabolic energy to regain competitive positions. This analogy underscores the strategic importance of early-race dynamics in sports where pacing, positioning, and tactical decision-making interact to shape performance outcomes.

Given these considerations, the present study aimed to identify the key variables influencing performance across different wind conditions in Olympic RS:X sailors using GPS-derived metrics. Based on previous evidence, it was hypothesized that both VMG and early-race positioning would be strongly associated with overall race performance.

## 2. Materials and Methods

### 2.1. Participants

The study sample consisted of Olympic RS:X class sailors (26 women and 28 men) who participated in the selected regatta. The age range (19–36 years for men and 19–38 years for women) reflects the actual ages of the competitors in the event and was not predetermined by the authors.

All participants included in the analysis were sailors competing in the RS:X class races of the selected event for whom complete GPS data were publicly available. No additional inclusion or exclusion criteria were applied. The data were obtained from official World Sailing sources [15]. This study is a secondary analysis of publicly available data obtained from official World Sailing sources. No data were independently collected by the authors.

### 2.2. Regatta

The analyzed event was the 2019 Hempel World Cup Series Final, held in Marseille, France, from 3’9 June 2019. This event was selected due to its high competitive level as an official World Sailing competition and because it provided one of the most complete publicly available datasets in terms of the number of races and GPS-derived performance variables. The racecourse comprised six legs: two upwind, two beam reach (sailing approximately perpendicular to the wind), and two downwind. Accordingly, each race was structured into sequential legs, and variables such as velocity made good (VMG), distance covered (m), and number of maneuvers were calculated separately for each leg. For clarity, these were labeled as the first (1st) and second (2nd) occurrences of each leg type (e.g., 1st upwind leg, 2nd upwind leg), which are reported in the Section 3.

The variables first-leg ranking, VMG (knots), distance covered (m) and number of maneuvers (i.e., the number of tacks and jibes performed during each race segment) were recorded using the SAP-Sailing^®^ application Walldorf, Germany [16]. This system operates with a TracTrac^®^ GPS device (TracTrac, Lyngby, Denmark) positioned on the sailor, with a sampling frequency of 5 Hz. The collected data were transmitted via mobile network and subsequently processed by the application. First-leg ranking was defined as the position of each sailor at the end of the first upwind leg, corresponding to the moment at which the sailors reached the first mark of the racecourse. This variable was obtained directly from the SAP-Sailing^®^ system based on GPS positional data. VMG was defined as the component of a sailor’s velocity in the direction of the next mark of the racecourse. Therefore, VMG represents effective progress toward the target mark for each race segment. In upwind legs, sailors progress against the wind toward the windward mark, whereas in downwind legs they sail with the wind toward the leeward mark. In beam reach legs, sailors travel approximately perpendicular to the wind direction, and VMG represents the velocity component toward the next mark despite sailing at an angle to the wind. All VMG values were obtained directly from the SAP-Sailing^®^ system based on GPS positional data. The values reported for maneuvers represent mean values across races; therefore, decimal values (e.g., 2.5) indicate the average number of maneuvers performed rather than discrete counts.

The average values of first-leg ranking, VMG, distance and maneuvers were obtained from the total number of races performed in the regatta. A total of 18 races were analyzed: 9 in the men’s group and 9 in the women’s group. Wind speed ranges in the races were 8.4–20.7 knots and 8.1–21.1 in the men’s and women’s groups, respectively.

Wind speed was classified for each race according to the categories established by the Royal Yachting Association [17]: >8 to ≤12 knots (light wind), >12 to ≤15 knots (moderate wind), and >15 knots (strong wind).

Information regarding additional environmental conditions such as wave height or sea state was not available from the official data sources and therefore could not be included in the analysis. This should be considered when interpreting the results.

### 2.3. Statistical Analyses

The data are presented as the mean (M) ± standard deviation (SD). All statistical tests were performed using IBM SPSS Statistics (version 20.0 for Mac, IBM Corp., Chicago, IL, USA). The values obtained for the variables were recorded for each of the six legs conducted in every race. The unit of analysis was defined at the race level. For each race, mean values of the analyzed variables (VMG, distance covered, and number of maneuvers) were calculated across sailors within each group (men and women). Races were classified according to wind conditions (light, moderate, and strong wind) based on the average wind speed recorded in each race. Statistical analyses were performed using these aggregated race-level values within each wind category. Because the data were aggregated at the race level, each observation was treated as independent within each wind condition category, and no repeated-measures structure was modeled. The same sailors generally participated across the races analyzed within each group. However, because the analyses were conducted using aggregated race-level mean values under different environmental conditions, each race was treated as an independent observational unit. Nevertheless, the potential influence of repeated participation across races should be considered when interpreting the findings. Pearson correlation coefficients were used to examine the association between predictor variables and performance. Predictor variables included the first-leg ranking, VMG, distance and number of maneuvers across upwind, beam reach and downwind courses [18]. Subsequently, multiple linear regression analyses were performed including variables with correlation coefficients greater than 0.3 [19]. Race performance (final ranking) was used as the dependent variable. The forward stepwise method was applied for model selection. The same analyses were conducted to investigate the association between the predictor variables and first-leg ranking. VMG, distance covered, and the number of maneuvers across the upwind, beam reach, and downwind legs were included as predictor variables.

## 3. Results

A total of 54 Olympic class sailors were studied. All GPS data were publicly accessible. Finally, GPS data from 18 races (9 for women) of RS:X class sailors were analyzed.

Table 1 presents the mean values of first-leg ranking, VMG, distance, and maneuvers for the men’s and women’s groups across the three wind conditions in each of the nine races analyzed in the regatta.

Table 2 shows the correlation coefficients between ranking in the race and the predictor variables across light, moderate, and strong wind conditions in the men’s and women’s groups. In the men’s group, the measurement showed significant correlations, with first-leg ranking and 1st VMG upwind under all wind conditions. These results were also observed in the women’s group.

Table 3 presents the correlation coefficients between first-leg ranking and the predictive variables under light, moderate, and strong wind conditions in the men’s and women’s groups. In both the men’s and women’s groups, 1st VMG upwind showed significant correlations under all wind conditions.

Table 4 shows the results of the multiple linear regression analysis using the forward stepwise method for the men’s group across light, moderate, and strong wind conditions, with ranking in the race as the dependent variable. The model obtained in light wind conditions showed a linear relationship of 87% and goodness-of-fit or R^2^ = 0.76. The value of Durbin–Watson (1.62) indicates that our error variance is independent; therefore, it would be appropriate to use a linear model. This model includes constant, first-leg ranking, 1st VMG upwind, 2nd VMG upwind and 1st VMG broad reach. For moderate wind conditions, the model obtained showed a linear relationship of 85% and goodness-of-fit or R^2^ = 0.73 (Durbin–Watson = 1.60). The model obtained include the following variables: constant, first-leg ranking, 1st VMG upwind, 2nd VMG upwind and 2nd VMG beam reach. In strong wind conditions, the result showed a linear relationship of 80% and goodness-of-fit or R^2^ = 0.64. The value of Durbin–Watson (1.53) indicates that our error variance is independent. This model includes constant, first-leg ranking and 2nd VMG beam reach.

Table 5 presents the results of the multiple linear regression analysis using the forward stepwise method for the women’ group across light, moderate, and strong wind conditions, with ranking in the race as the dependent variable. The model obtained in light wind conditions showed a linear relationship of 88% and goodness-of-fit or R^2^ = 0.78 (Durbin–Watson = 1.50). This model includes constant, first-leg ranking, 1st VMG upwind, 2nd VMG broad reach and 2nd VMG beam reach. For moderate wind conditions, the model obtained showed a linear relationship of 0.84% and goodness-of-fit or R^2^ = 0.72. The value of Durbin–Watson was 1.95. The variables included in this model were constant and first-leg ranking. In strong wind conditions, the result showed a linear relationship of 82% and goodness-of-fit or R^2^ = 0.67. The value of Durbin–Watson was 1.63. This model includes constant, first-leg ranking and 2nd VMG upwind.

Table 6 shows the results of the multiple linear regression analysis using the forward stepwise method for the men’s group across light and strong wind conditions, with first-leg ranking as the dependent variable. For light wind conditions, the model obtained showed a linear relationship of 64% and goodness-of-fit or R^2^ = 0.42 (Durbin–Watson = 1.17). The variables of this model were constant and 1st VMG upwind. In strong wind conditions, the model obtained showed a linear relationship of 76% and goodness-of-fit or R^2^ = 0.58. The value of Durbin–Watson (2.01) indicated that the error variance was independent; therefore, the use of a linear model was considered appropriate. This model includes constant, 1st VMG upwind, 1st distance upwind and 1st maneuvers upwind.

Table 7 displays the forward stepwise multiple linear regression results for the women’s group, using first-leg ranking as the dependent variable under light, moderate, and strong wind conditions. In light wind conditions, the model obtained showed a linear relationship of 51% and goodness-of-fit or R^2^ = 0.27. The value of Durbin–Watson was 1.34. This model includes constant and 1st VMG upwind. For moderate wind conditions, the result showed a linear relationship of 64% and goodness-of-fit or R^2^ = 0.41. The value of Durbin–Watson (1.84) indicates that our error variance is independent. The variables included in this model were constant, 1st VMG upwind and 1st VMG broad reach. In strong wind conditions, the results showed a linear relationship of 60% and goodness-of-fit or R^2^ = 0.35 (Durbin–Watson = 1.34). This model includes constant and 1st VMG upwind.

## 4. Discussion

The present study investigated the biomechanical and tactical factors associated with performance in Olympic RS:X sailing across a broad spectrum of wind conditions, using high-frequency GPS data to quantify VMG, the distance covered, maneuver frequency, and in-race positioning. Across all analyses, first-leg ranking was consistently the variable most strongly associated with the final race outcome for both men and women, independent of wind intensity. This finding aligns with previous research indicating that early tactical positioning exerts a disproportionate influence on subsequent race dynamics, particularly in classes where course geometry, windward congestion, and limited overtaking lanes constrain tactical flexibility [9]. The strength of these associations in the present dataset (r = 0.75–0.84) supports the important role of the opening upwind leg in relation to competitive trajectories.

A second major finding concerns the central importance of VMG during the first upwind leg, which emerged as the technical variable most strongly associated with performance across nearly all wind categories. Upwind VMG integrates multiple performance components, tactical decision-making, hydrodynamic efficiency, sail trim, and pumping effectiveness, making it a comprehensive indicator of technical proficiency. The stronger correlations observed in women suggest that performance variability in this group may be more sensitive to upwind efficiency, potentially reflecting differences in body mass, sail-handling strategies, or risk-taking behavior compared with men [20,21,22].

Wind intensity modulated the relative importance of specific performance indicators. Under medium winds, both men and women relied heavily on VMG in upwind and downwind legs, consistent with the mixed metabolic demands of pumping and transitions between displacement and planning modes. In very strong winds, however, VMG during the beam reach was strongly associated with performance, particularly among men. This shift reflects the hydrodynamic characteristics of the RS:X board, which achieves its highest speeds on reaching legs when fully powered; under these conditions, small differences in board trim, balance, and control can produce disproportionately large performance gains [3,23]. The results also highlight the analytical value of GPS-derived metrics for performance assessment in elite sailing. VMG, distance, and maneuver counts provided objective, high-resolution insights into tactical and biomechanical strategies, reinforcing the utility of GPS-based analytics for identifying key performance-related indicators. The consistency of the regression models across wind categories further supports the robustness of these indicators and their relevance for applied performance monitoring (Table 4, Table 5, Table 6 and Table 7).

The parallel drawn with middle-distance running underscores a broader principle of race strategy: early positioning is associated with the probability of success. Just as the first 200 m of an 800 m race are associated with tactical freedom and metabolic efficiency, the first upwind leg in RS:X racing is associated with the tactical landscape for the remainder of the race [11,12,13,14,24]. Athletes who secure a strong early position reduce exposure to disturbed airflow, minimize tactical constraints, and preserve physiological resources for later segments.

Future research should explore whether integrating physiological data, such as heart rate, power output, or sail-pumping frequency, with GPS metrics could further clarify how athletes manage the interplay between biomechanics, tactics, and fatigue across varying wind conditions. Such multimodal datasets may enhance the predictive value of performance models and provide deeper insight into the physiological cost of tactical decisions.

Several limitations should be considered when interpreting the findings. First, the use of publicly available GPS data restricted the analysis to the variables provided by the SAP-Sailing^®^ platform. Although the system offers high-frequency positional information, it does not include complementary biomechanical or physiological metrics, limiting the ability to fully characterize internal load or fatigue-related performance changes. Second, wind conditions were classified based on average race values; short-term fluctuations within each leg were not analyzed, potentially masking micro-tactical adaptations that influenced VMG and ranking. In addition, one regression model corresponding to the men’s group under moderate wind conditions was excluded from the final interpretation because the Durbin–Watson statistic indicated non-independence of the residuals (DW = 0.83), suggesting that the assumptions required for linear regression were not adequately satisfied. Additionally, some regression models showed Durbin–Watson values within the lower acceptable range (e.g., DW = 1.17–1.34), which may have indicated potential mild residual autocorrelation. Therefore, the robustness of the findings derived from these specific models should be interpreted with caution. Furthermore, although the analyses were conducted using aggregated race-level data, many of the same sailors competed repeatedly across races. Therefore, some degree of dependency between observations cannot be completely excluded, which should be considered when interpreting the results. Third, the study did not examine sex-specific biomechanical or tactical strategies beyond statistical comparisons. Although men and women competed on identical equipment, differences in body mass, strength, and technique may influence the VMG and maneuvering efficiency in ways not captured by the current variables. Addressing these aspects in future research could deepen the understanding of performance determinants in Olympic windsurfing and improve the precision of GPS-based analytics.

## 5. Conclusions

Across all wind categories and in both sexes, the first-leg position exhibited the strongest association with final race ranking, with correlation coefficients ranging from 0.75 to 0.84. The regression analyses confirmed that this variable alone accounted for the majority of the explained variance in performance, indicating that the competitive outcome was largely shaped during the opening upwind leg. This segment represents the phase of greatest tactical density, where fleet congestion, disturbed airflow, and restricted overtaking lanes amplify the strategic value of early positioning.

Upwind VMG during the first-leg emerged as the only technical variable consistently associated with both first-leg ranking and overall performance across all wind conditions. This finding establishes upwind VMG as the primary technical key performance indicator (KPI) in RS:X racing. Among women, the influence of this variable was even more pronounced, suggesting that upwind efficiency constituted the most sensitive and discriminative performance factor within this group.

These findings underscore the critical influence of early-race dynamics and upwind technical proficiency in determining competitive success within Olympic RS:X windsurfing. Consequently, training programs should prioritize technical development on the upwind leg, specifically emphasizing the initial beat, given its decisive impact on overall race outcomes.

## Figures and Tables

**Table 1:** Mean ± SD of the variables analyzed in women (*n* = 26) and men (*n* = 28) with light (>8 to ≤12 knots), moderate (>12 to ≤15 knots) and strong (>15 knots) wind.

	Light Wind		Moderate Wind		Strong Wind	
Variable	Women	Men	Women	Men	Women	Men
Age (years)	25.4 ± 5.7	25.4 ± 5.1	25.4 ± 5.7	25.4 ± 5.1	25.4 ± 5.7	25.4 ± 5.1
First-leg ranking	13.5 ± 7.5	14.7 ± 8.4	13.0 ± 7.2	14.5 ± 8.1	13.0 ± 7.2	14.3 ± 8.0
1st VMG upwind (knots)	2.6 ± 0.3	2.8 ± 0.3	4.6 ± 0.9	5.7 ± 0.6	5.4 ± 0.7	6.6 ± 0.7
1st distance upwind (m)	1334.7 ± 242.2	1522.9 ± 440	2868.7 ± 502.5	2583.3 ± 260.5	3042.1 ± 445.4	2733.4 ± 153.1
1st maneuvers upwind	4.0 ± 1.6	3.7 ± 1.5	2.5 ± 0.9	1.9 ± 0.7	3.6 ± 1.3	3.0 ± 1.3
1st VMG beam reach (knots)	7.7 ± 2.3	7.2 ± 2.2	17.2 ± 2.5	18.6 ± 1.3	5.1 ± 1.0	16.5 ± 2.9
1st distance beam reach (m)	609.1 ± 14.6	641.8 ± 50.3	737.4 ± 87.1	739.7 ± 10.1	2260.4 ± 430.2	1495.2 ± 457.1
1st maneuvers beam reach	0.2 ± 0.4	0.2 ± 0.4	0.2 ± 0.4	0.2 ± 0.4	3.2 ± 2.3	1.5 ± 1.3
1st VMG broad reach (knots)	3.9 ± 0.2	5.3 ± 1.5	9.5 ± 2.1	11.1 ± 1.5	17.5 ± 4.4	7.9 ± 2.6
1st distance broad reach (m)	793.3 ± 231.2	1240.4 ± 356.4	1389.9 ± 266.2	1754.7 ± 116.2	948.4 ± 736.2	2440.6 ± 454.9
1st maneuvers broad reach	6.2 ± 3.5	3.1 ± 1.5	1.9 ± 0.5	2.0 ± 0.5	0.4 ± 1.6	2.8 ± 1.2
2nd VMG upwind (knots)	2.3 ± 0.2	2.7 ± 0.3	4.8 ± 0.8	5.1 ± 1.1	11.8 ± 2.6	17.5 ± 7.0
2nd distance upwind (m)	848.9 ± 85.9	1202.1 ± 295.1	2038.4 ± 427.2	2846.1 ± 590.0	1389.7 ± 262.6	1371.4 ± 818.6
2nd maneuvers upwind	3.7 ± 1.3	3.3 ± 1.3	1.9 ± 0.6	2.8 ± 1.8	2.7 ± 1.2	0.5 ± 0.9
2nd VMG beam reach (knots)	5.1 ± 0.7	4.4 ± 0.4	15.4 ± 2.4	10.8 ± 2.3	17.4 ± 4.0	14.4 ± 2.1
2nd distance beam reach (m)	233.6 ± 21.9	1122.4 ± 433.3	311.1 ± 37.3	1744.4 ± 291.0	262.9 ± 54.3	1443.7 ± 255.3
2nd maneuvers beam reach	0.3 ± 0.5	3.4 ± 1.9	0.4 ± 0.4	1.2 ± 0.6	0.2 ± 0.4	2.4 ± 0.8
2nd VMG broad reach (knots)	4.3 ± 0.3	6.5 ± 1.7	10.1 ± 1.6	17.2 ± 3.8	12.6 ± 2.9	18.5 ± 2.8
2nd distance broad reach (m)	876.5 ± 217.3	233.2 ± 35.6	1752.7 ± 231.0	309.5 ± 42.3	1312.8 ± 289.3	282.3 ± 32.6
2nd maneuvers broad reach	5.6 ± 3.2	0.2 ± 0.4	2.2 ± 0.7	0.0 ± 0.1	2.7 ± 0.9	0.4 ± 0.5

Data presented as M ± SD. M: mean. SD: standard deviation. VMG: velocity made good.

**Table 2:** Correlation coefficients (*r*) between ranking in the race and predictive variables in women (*n* = 26) and men (*n* = 28) across light (>8–≤12 knots), moderate (>12–≤15 knots), and strong (>15 knots) wind conditions.

	Light Wind		Moderate Wind		Strong Wind	
Variable	Women	Men	Women	Men	Women	Men
First-leg ranking	0.78 **	0.75 **	0.84 **	0.76 **	0.79 **	0.78 **
1st VMG upwind (knots)	−0.51 **	−0.48 **	−0.43 **	−0.53 **	−0.60 **	−0.57 **
1st distance upwind (m)	0.19	−0.20	−0.31	0.48 **	0.22	0.51 **
1st maneuvers upwind	0.25 *	0.11	0.15	0.29 *	0.02	0.34 **
1st VMG beam reach (knots)	−0.25 *	−0.24 *	−0.20	−0.30 *	−0.28 *	−0.24 **
1st distance beam reach (m)	0.00	−0.68	−0.05	−0.09	0.10	0.06
1st maneuvers beam reach	0.18	−0.19	−0.07	−0.1	0.17	−0.02
1st VMG broad reach (knots)	−0.50 **	−0.32 **	−0.33 **	−0.24	0.51 *	−0.21 *
1st distance broad reach (m)	0.36 **	0.12	0.02	0.15	−0.06	0.06
1st maneuvers broad reach	−0.00	−0.04	−0.06	−0.08	0.25 *	0.12
2nd VMG upwind (knots)	0.07	−0.67 **	−0.28 *	−0.42 **	−0.48 **	−0.10
2nd distance upwind (m)	0.05	−0.06	−0.21	0.19	0.10	0.02
2nd maneuvers upwind	0.06	0.24 *	−0.04	0.36 **	0.20	−0.02
2nd VMG beam reach (knots)	−0.35 **	−0.00	−0.21	0.33 *	−0.39 **	−0.46 **
2nd distance beam reach (m)	−0.03	−0.02	−0.10	−0.10	−0.24 *	0.02
2nd maneuvers beam reach	0.13	−0.29 **	−0.16	−0.08	−0.18	0.17
2nd VMG broad reach (knots)	−0.36 **	−0.18	−0.10	−0.30 *	−0.32 **	−0.38 **
2nd distance broad reach (m)	0.04	−0.05	−0.21	−0.25	−0.32 **	−0.12
2nd maneuvers broad reach	0.07	0.08	−0.09	0.07	0.12	−0.11

VMG: velocity made good. * *p* ≤ 0.05. ** *p* ≤ 0.01.

**Table 3:** Correlation coefficients (*r*) between first-leg ranking and predictor variables in women (*n* = 26) and men (*n* = 28) across light (>8–≤12 knots), moderate (>12–≤15 knots), and strong (>15 knots) wind conditions.

	Light Wind		Moderate Wind		Strong Wind	
Variable	Women	Men	Women	Men	Women	Men
1st VMG upwind (knots)	−0.63 **	−0.64 **	−0.49 **	−0.65 **	−0.71 **	−0.68 **
1st distance upwind (m)	0.10	−0.04	0.10	0.62 **	0.30 **	0.62 **
1st maneuvers upwind	0.18	−0.02	0.10	0.39 **	0.07	0.50 **
1st VMG beam reach (knots)	−0.06	−0.17	−0.18	−0.24	−0.23 *	−0.18
1st distance beam reach (m)	0.01	−0.00	−0.07	−0.20	0.13	−0.08
1st maneuvers beam reach	0.14	0.05	−0.17	−0.03	0.19	−0.09
1st VMG broad reach (knots)	−0.28 *	−0.18	−0.33 **	−0.16	−0.41 **	−0.13
1st distance broad reach (m)	0.21	0.11	0.05	0.12	−0.19	−0.03
1st maneuvers broad reach	−0.15	−0.12	−0.03	−0.25	0.22	−0.00
2nd VMG upwind (knots)	0.11	−0.44 **	−0.26 *	−0.25	−0.35 **	−0.10
2nd distance upwind (m)	0.18	−0.04	−0.21	0.06	−0.03	0.01
2nd maneuvers upwind	0.10	0.20	−0.06	0.21	−0.05	−0.01
2nd VMG beam reach (knots)	−0.07	0.14	−0.23 *	−0.25	−0.29 **	−0.38 **
2nd distance beam reach (m)	−0.07	−0.00	−0.11	−0.09	−0.30 **	−0.05
2nd maneuvers beam reach	0.02	−0.29 **	−0.12	−0.14	−0.15	0.07
2nd VMG broad reach (knots)	−0.20	0.14	−0.09	−0.26 *	−0.25 *	−0.31 **
2nd distance broad reach (m)	0.02	−0.06	−0.23 *	−0.19	−0.30 **	−0.12
2nd maneuvers broad reach	0.04	0.07	−0.09	−0.02	−0.24 *	−0.07

VMG: velocity made good. * *p* ≤ 0.05. ** *p* ≤ 0.01.

**Table 4:** Linear regression coefficients for the men’s group with ranking in the race as the dependent variable.

Model		Non-Standardized Coefficients		Standardized Coefficients	t	Sig.	Confidence Interval 95% for *B*	
		*B*	Std. Error	*β*			Lower Bound	Upper Bound
Light wind	Constant	−1.13	9.92		−0.11	0.09	−20.88	18.60
	First-leg ranking	0.85	0.09	0.86	8.55	<0.001	0.65	1.04
	1st VMG upwind	13.23	3.35	0.54	3.95	<0.001	6.56	19.90
	2nd VMG upwind	−9.73	1.88	−0.44	−5.17	<0.001	−13.48	−5.99
	1st VMG broad reach	−1.42	0.67	−0.27	−2.12	0.037	−2.76	−0.08
Moderate wind	Constant	−22.78	9.78		−2.32	0.02	−42.43	−3.13
	First-leg ranking	1.00	0.11	1.00	9.12	<0.001	0.78	1.23
	1st VMG upwind	7.31	2.00	0.58	3.64	<0.001	3.28	11.35
	2nd VMG upwind	−7.58	1.54	−1.07	−4.91	<0.001	−10.68	−4.48
	2nd VMG beam reach	1.81	0.63	0.51	2.87	0.006	0.54	3.08
Strong wind	Constant	15.01	3.83		3.91	<0.001	7.41	22.61
	First-leg ranking	0.71	0.06	0.70	11.33	<0.001	0.58	0.83
	2nd VMG beam reach	−0.75	0.23	−0.20	−3.21	<0.001	−1.21	−0.28

Note: Method used: forward stepwise. Statistically significant association. *B* = linear regression coefficient. *β* = standardized partial regression coefficient. Std: standard. VMG: velocity made good. Light wind: >8–≤12 knots. Moderate wind: >12–≤15 knots. Strong wind: >15 knots.

**Table 5:** Linear regression coefficients for the women’s group with ranking in the race as the dependent variable.

Model		Non-Standardized Coefficients		Standardized Coefficients	t	Sig.	Confidence Interval 95% for *B*	
		*B*	Std. Error	*β*			Lower Bound	Upper Bound
Light wind	Constant	86.60	12.75		6.79	<0.001	61.16	112.05
	First-leg ranking	0.45	0.08	0.45	5.30	<0.001	0.28	0.62
	1st VMG upwind	−8.68	1.91	−0.40	−4.54	<0.001	−12.49	−4.87
	1st VMG broad reach	−4.39	2.05	−0.15	−2.13	0.03	−8.49	−0.29
	2nd VMG broad reach	−6.12	1.67	−0.27	−3.66	<0.001	−9.45	−2.78
	2nd VMG beam reach	−2.31	0.68	−0.22	−3.39	0.001	−3.67	−0.95
Moderate wind	Constant	1.86	0.93		1.99	0.05	−0.00	3.72
	First-leg ranking	0.86	0.06	0.84	13.74	0.000	0.73	0.98
	Constant	10.88	2.98		3.65	0.000	4.94	16.83
Strong wind	First-leg ranking	0.70	0.07	0.70	9.51	0.000	0.56	0.85
	2nd VMG upwind	−0.59	0.20	−0.21	−2.94	0.004	−1.00	−0.19

Note: Method used: forward stepwise. Statistically significant association. *B* = linear regression coefficient. *β* = standardized partial regression coefficient. Std: standard. VMG: velocity made good. Light wind: >8–≤12 knots. Moderate wind: >12–≤15 knots. Strong wind: >15 knots.

**Table 6:** Multiple linear regression coefficients for the men’s group with first-leg ranking as the dependent variable under light and strong wind conditions.

Model		Non-Standardized Coefficients		Standardized Coefficients	t	Sig.	Confidence Interval 95% for *B*	
		*B*	Std. Error	*β*			Lower Bound	Upper Bound
Light wind	Constant	59.51	5.85		10.15	<0.001	47.86	71.17
	1st VMG upwind	−15.94	2.07	−0.64	−7.70	<0.001	−20.05	−11.82
Strong wind	Constant	−4.34	16.62		−0.26	0.79	−37.28	28.60
	1st VMG upwind	−4.05	0.90	−0.39	−4.50	<0.001	−5.84	−2.26
	1st distance upwind	0.01	0.00	0.28	3.33	0.001	0.00	0.02
	1st maneuvers upwind	1.68	0.40	0.28	4.21	<0.001	0.89	2.48

Note: Method used: forward stepwise regression *B* = linear regression coefficient. *β* = standardized partial regression coefficient. VMG: velocity made good. Light wind: >8–≤12 knots. Strong wind: >15 knots.

**Table 7:** Linear regression coefficients for the women’s group with first-leg ranking as the dependent variable.

Model		Non-Standardized Coefficients		Standardized Coefficients	t	Sig.	Confidence Interval 95% for *B*	
		*B*	Std. Error	*β*			Lower Bound	Upper Bound
Light wind	Constant	42.80	5.69		7.51	<0.001	31.54	54.15
	1st VMG upwind	−11.12	2.14	−0.51	−5.19	<0.001	−15.40	−6.85
Moderate wind	Constant	27.88	3.27		8.51	<0.001	21.35	34.41
	1st VMG upwind	−11.62	1.72	−1.51	−6.72	<0.001	−15.07	−8.18
	1st VMG broad reach	4.06	0.77	1.18	5.24	<0.001	2.52	5.61
Strong wind	Constant	42.80	4.70		9.10	<0.001	33.44	52.17
	1st VMG upwind	−5.51	0.86	−0.60	−6.40	<0.001	−7.22	−3.79

Note: Method used: forward stepwise. Statistically significant association. *B* = linear regression coefficient. *β* = standardized partial regression coefficient. Dependent variable: first-leg ranking. Std: standard. VMG: velocity made good. Light wind: >8–≤12 knots. Moderate wind: >12–≤15 knots. Strong wind: >15 knots.

## Data Availability

The data used in this study are publicly available from the official World Sailing^®^ and SAP-Sailing^®^ platforms.
